# Ionizing Radiation Detectors Based on Ge-Doped Optical Fibers Inserted in Resonant Cavities

**DOI:** 10.3390/s150204242

**Published:** 2015-02-12

**Authors:** Saverio Avino, Vittoria D’Avino, Antonio Giorgini, Roberto Pacelli, Raffaele Liuzzi, Laura Cella, Paolo De Natale, Gianluca Gagliardi

**Affiliations:** 1 Consiglio Nazionale delle Ricerche, Istituto Nazionale di Ottica (INO), via Campi Flegrei 34—Comprensorio A. Olivetti, 80078 Pozzuoli (Na), Italy; E-Mails: antonio.giorgini@ino.it (A.G.); gianluca.gagliardi@ino.it (G.G.); 2 Consiglio Nazionale delle Ricerche, Istituto di Biostrutture e Bioimmagini, via Tommaso De Amicis 95, 80131 Napoli, Italy; E-Mails: vittoria.davino@ibb.cnr.it (V.D.); raffaele.liuzzi@cnr.it (R.L.); laura.cella@cnr.it (L.C.); 3 Università di Napoli Federico II, Dipartimento di Scienze Biomediche Avanzate, via Pansini 5, 80131 Napoli, Italy; E-Mail: pacerto@yahoo.com; 4 Consiglio Nazionale delle Ricerche, Istituto Nazionale di Ottica (INO), Largo Enrico Fermi 6, 50125 Firenze, Italy; E-Mail: paolo.dentale@ino.it

**Keywords:** ionizing radiation, optical fiber sensors, optical resonators, optical materials

## Abstract

The measurement of ionizing radiation (IR) is a crucial issue in different areas of interest, from environmental safety and industrial monitoring to aerospace and medicine. Optical fiber sensors have recently proven good candidates as radiation dosimeters. Here we investigate the effect of IR on germanosilicate optical fibers. A piece of Ge-doped fiber enclosed between two fiber Bragg gratings (FBGs) is irradiated with gamma radiation generated by a 6 MV medical linear accelerator. With respect to other FBG-based IR dosimeters, here the sensor is only the bare fiber without any special internal structure. A near infrared laser is frequency locked to the cavity modes for high resolution measurement of radiation induced effects on the fiber optical parameters. In particular, we observe a variation of the fiber thermo-optic response with the radiation dose delivered, as expected from the interaction with Ge defect centers, and demonstrate a detection limit of 360 mGy. This method can have an impact in those contexts where low radiation doses have to be measured both in small volumes or over large areas, such as radiation therapy and radiation protection, while bare optical fibers are cheap and disposable.

## 1. Introduction

In the last decades, there has been a growing need for detection and quantification of IR in different contexts, such as environmental safety, industrial processes monitoring, radiation protection and medicine. In this regard, radiation dosimetry plays a central role to quantify the absorbed energy and thus assess radiation effects. When IR is applied in medical settings, the main issue is the measurement of the amount of radiation that is actually delivered to the patient [1]. The need for accurate dosimetry is greatest in radiation therapy for cancer. One of the aspects of an efficient treatment of cancer is the calibration of the clinical accelerator machines (reference dosimetry), but also accurate radiation beam shaping and measurement of dose delivered to the patient (relative and *in vivo* dosimetry) must be taken into account [2–5]. A number of physical and chemical devices based on liquids, solids and gases are currently available to detect IR and measure the released dose. Ionization chambers, silicon diodes, thermoluminescence devices, MOSFETs, diamond detectors and films are currently adopted as reference, relative and *in vivo* dosimeters [6–9]. However, among the above mentioned sensors, none meet the major requirements of an ideal dosimeter, which should be small in size but highly sensitive. The sensing element should only minimally perturb the radiation field and always measure the total absorbed dose. Moreover, immunity to environmental conditions and external interferences are desirable. Air-filled ionization chambers (sensitive volumes 0.6 cm^3^), for instance, have been the standard detectors for radiotherapy dosimetry, but they are not always suitable when high dose gradients (small fields), high dose-per-pulse, time-dose variance, and non-uniform beam distributions occur [10]. Volume averaging and lack of electronic equilibrium, which require a sufficiently large region of beam uniformity around the detector, complicate the use of ionization chambers in applications requiring high spatial resolution. A higher spatial resolution can be obtained with solid state detectors like diodes or diamonds due to their high sensitivity, and accordingly they can be realized with smaller dimensions (10^−4^–10^−3^ cm^3^). Liquid-filled ionization chambers with reduced sizes (0.002 cm^3^) have been also introduced in high precision radiation dosimetry. Liquid filled ionization chambers, like solid state detectors, combine a very small volume with a relatively high response, but further studies on the underlying theories for recombination effects are still needed for them to become a feasible option.

Recently, a considerable effort has been spent to demonstrate the feasibility of optical fiber sensing for application in radiation environments, although only a few experimental proofs have been provided on this subject [11]. So far, conventional FBG sensors, which have been successfully used for temperature, strain, and pressure monitoring [12], have been proposed for IR detection. FBG-based dosimeters transmit dose information through the fiber. Consequently, these sensors are immune to electromagnetic interference, which is usually a serious issue for electronic dosimeters. The ability to remotely monitor radiation is an additional advantage, as the sensor can be placed at a long distance from the readout electronics. Also, optical fiber sensors can be multiplexed so that a single reading unit can control several sensors. Thanks to their mechanical nature and very small size, fibers lend themselves to the realization of miniature radiation detectors with different geometries without perturbing the dose measurement. Optical sensors are generally based on either internal generation of light caused by radiation-matter interaction or changes of intrinsic physical-chemical properties of the material itself [13]. Most existing optical fiber-based dosimeters are extrinsic sensors. In this case, the role of fibers is primarily to transmit light from a thermoluminescent solid, a diode or a scintillating medium [13–15]. Recently, doped optical fibers have also been investigated as radioluminescent dosimeters [16,17]. The main drawback of such a system relies in the so-called “stem effect”, *i.e.*, unwanted light contribution deriving from Cerenkov radiation generated in the fiber, which affects the reliability of dose measurements. There are also a few studies that demonstrate radiation-induced attenuation within a fiber. One of the underlying mechanisms is the creation of defect centers by interaction of high-energy photons with OH and Ge, naturally present in silica optical fibers, as well as with added dopants. The effects of ionizing radiation have been found to be sufficient to induce losses much greater than intrinsic fiber loss (∼dB/km) from the visible to the near-infrared spectral range, even at low/moderate doses (in the range 1–10 Gy) [18–20]. In all these cases, the interaction with IR can be quantified by measurements of the transmitted light intensity. Besides the increase in optical transmission loss, a correlated change of average refractive index also occurs in the optical waveguide, which can be exploited to extract the desired information with very high resolution [21,22]. Refractive sensing has the advantage of overcoming typical issues related to radiation power measurements, as well as systematic deviations due to fiber instability (bending, stress, degradation, *etc.*) that also limit repeatability and long-distance transmission for remote readout.

In a previous work [22] we demonstrated that a stand-alone phase-shifted FBG grating can be efficiently used as a passive dosimeter. In particular, we exploited the fact that after exposure to IR there is a change of both the refractive index and thermo-optic response of the fiber grating itself. In this article, after a review of theoretical models and phenomenology related to radiation induced effects on Ge-doped optical fiber, we show that an optical fiber resonator formed by two identical FBGs as end mirrors may serve as a high sensitivity detector for low dose IR measurements. In particular, as sensing region we use a portion of the Ge-doped fiber enclosed between the two FBGs, which are not exposed to IR but are only used as cavity reflectors. After irradiation, we observe a variation of the overall cavity thermo-optic response, which is directly related to the dose delivered to the fiber. In order to measure the thermo-optic response, we frequency lock a laser to the cavity and then monitor the wavelength shift of its resonance modes with the temperature. We demonstrate a detection limit of 360 mGy.

## 2. Phenomenology and Structural Models of Radio-Induced Point Defects in Ge Doped Optical Fiber

Exposure to IR creates damage to the structure of the irradiated material. Many authors have published on this subject and identified the physical and chemical mechanisms that induce effects on the silicon dioxide network [23–27]. Different types of structural models have been proposed to explain the photosensitivity of Ge doped silica. The first class of such models is termed “*color center model*” and is based on the generation or the conversion of point defects. The defects cause an alteration of the spectroscopic properties of vitreous silica, with occurrence of absorption bands that, through the Kramers-Kronig relations, provide an explanation for the refractive index variation [25,28]. Other authors propose models predicting “*compaction effects*” as the origin of the refractive index variations and they describe this variation through the Lorentz-Lorentz relation [29,30]. There are also some models that mix the *color centers* and the *compaction effects* models [31]. In this case, the conversion of a type of defect into another involves a large network reorganization, which originates the densification and, as a consequence, the refractive index variation.

The point defects are generated through two mechanisms named knock-on and radiolysis processes. The knock-on process consists in the impact between the bullet and an atom of the glass network which absorbs sufficient kinetic energy to move from its original position. In the case of silica, in order to create a defect through a knock-on, an energy of 10 eV is necessary for an oxygen atom and 20 eV for a silicon atom, the Si-O bond energy being about 5 eV. The radiolysis process consists in the ionization and electronic excitations induced by photons, which later may lead to the formation of point defects. Most of the electron-hole pairs generated by the radiolysis process give rise to luminescence through their recombination, while the remaining pairs interact with phonons and may cause the displacement of some atoms from their original position or can break some bonds [32]. The presence of impurities of Ge-atoms facilitates the formation of defects in the silica matrix: since the energy of the Ge-O bond (∼3.6 eV [33]) is lower than that of the Si-O bond, the breaking of these bonds is more probable with respect to that of the Si-O [34]. Many researchers have studied Ge-doped silica samples by investigating paramagnetic defects through electron paramagnetic resonance spectroscopy [24–27,35–37]. Diamagnetic defects can also be directly investigated by photoluminescence (PL) or cathodoluminescence spectroscopy [38,39]. The point defects are classified into intrinsic defects when the atomic species constituting the material are involved (O or Si atoms), or extrinsic defects when chemically different atoms such as Ge atoms are involved.

**Figure 1.**
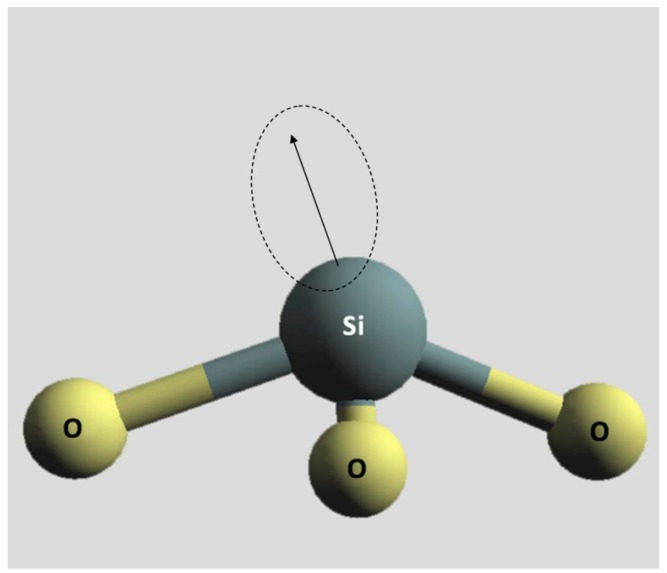
Schematic illustration of a generic E′-center.

**Figure 2.**
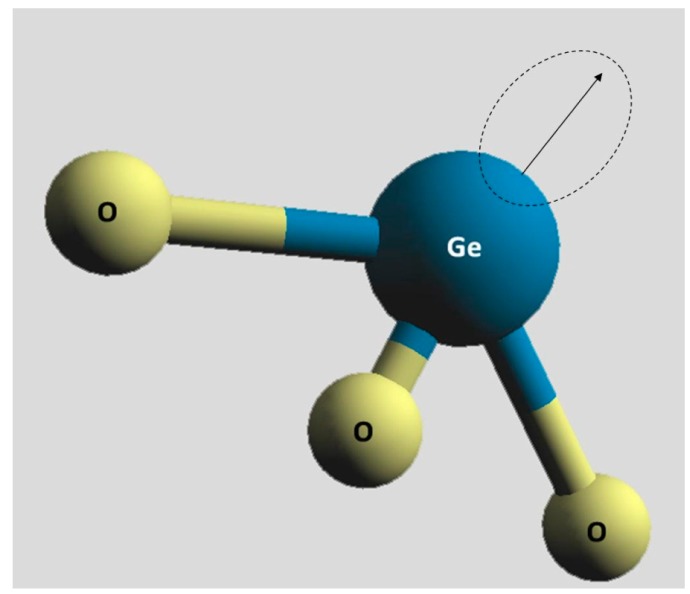
Schematic illustration of a generic GeE′-center.

There are several intrinsic defects in Ge doped silica. The E′-center, which is generally denoted by the symbol ≡Si•, where the three parallel lines represent three oxygen separate bonds to one silicon atom and the dot denotes the unpaired electron (Figure 1). The oxygen-deficiency center (ODC) which is a neutral oxygen vacancy (Neutral Oxygen Vacancy–NOV), and can be divided into: the relaxed neutral oxygen vacancy ODC(I), denoted as ≡Si—Si≡, the unrelaxed neutral oxygen vacancy, denoted as ≡Si⋯Si≡, and the twofold coordinated silicon (Silicon Lone Pair Center–SLPC), denoted as =Si•• ODC(II). The non-bridging oxygen hole center (NBOHC), denoted as ≡Si–O•, which can be visualized as the oxygen part of a broken bond. The peroxy bridge (POL), denoted as ≡Si–O–O–Si≡, which is formed by oxygens in excess which create oxygen-oxygen bonds. The peroxy radical (POR) denoted as ≡Si–O–O•, which shows a paramagnetic defect with a O–O bond in the silica structure and the presence of interstitial oxygen atoms. There are also various extrinsic defects: the GeE′, denoted as ≡Ge•, which is structurally identical to the E′ center apart from substitution of Si with Ge (Figure 2): the two fold coordinated Germanium (GeODC(II)) or Germanium Lone Pair Center (GLPC), denoted as =Ge•• and the neutral oxygen vacancy (NOV), denoted as ≡Ge⋯Si≡; the Ge(1) and Ge(2) (Germanium Electron Center), denoted as ≡GeO4•, which are electron traps constituted by an electron trapped on a tetracoordinated Ge atom with a Si or Ge atom in the adjacent tetrahedron, respectively. A more comprehensive review of both the nature and the molecular structure of radiation-induced point defects in pure and doped glassy silica can be found in [27,36,37].

## 3. Experimental Section and Results

**Figure 3.**
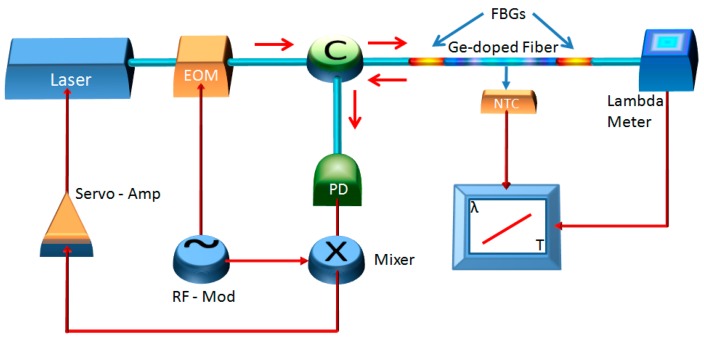
Experimental apparatus scheme. A DFB laser emitting at 1560 nm is frequency locked to an optical fiber cavity made by two FBGs. Variations of the thermo-optic response of the optical fiber forming the cavity are measured by scanning the temperature and measuring the wavelength of the light resonating into the cavity. EOM: Electro-optic modulator; C: Optical fiber circulator; PD: Photodiode; RF-Mod: Radio frequency modulation; NTC: Negative temperature coefficient resistor; Servo-Amp: Filtering and amplifying.

The detection method relies on the measurement of the thermo-optic response variation of a short length of Ge-doped optical fiber after exposure to IR. With respect to wavelength shift measurements, which are usually affected by environmental noise, such as thermal drifts and mechanical instabilities, the measurement of the thermo-optic response is substantially immune to these perturbations [22]. The sensing region (2-cm long) is enclosed between two FBGs. The FBGs form an optical cavity which is interrogated by a near-infrared laser. The laser is frequency locked to a cavity mode using a Pound-Drever-Hall scheme [40]. By scanning the fiber temperature and monitoring the laser wavelength, the thermo-optic response is retrieved. The experimental apparatus is shown in Figure 3.

A 10 mW DFB Er-fiber laser emitting around 1560 nm (Koheras Adjustik ^TM^, Birkerød, Denmark) is phase modulated at 72 MHz by an all-fiber electro-optic modulator. After modulation two sidebands are generated and overlapped to the laser carrier. This triplet enters the first port of a fiber circulator and then injects the FBG-cavity. When the cavity is on resonance, the sidebands are reflected back by the first FBG and interfere with the carrier resonating into the cavity.

The whole reflected field beam exits the second port of the circulator and is detected by a fast (2 GHz) InGaAs photodetector. The detector signal is demodulated with the 72 MHz modulation signal in a radiofrequency mixer, yielding an error signal, centered around the cavity mode, which is filtered and amplified by a servo system. The servo signal is finally fed back to the laser current, in order to lock its frequency to a cavity mode.

The FBG cavity is inserted into a thermally controlled chamber whose temperature is finely changed around the ambient value (25 °C), the fiber temperature being monitored by a negative temperature coefficient (NTC) resistor placed in contact with the fiber. The cavity transmitted light is collected by a high resolution lambda-meter that measures the center wavelength of the resonating cavity mode in real-time.

**Figure 4.**
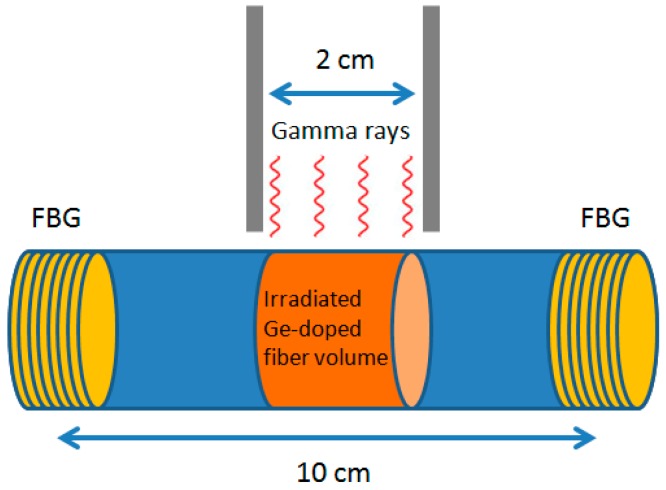
Irradiation of a Ge-doped optical fiber enclosed between two FBGs with gamma rays from a 6 MV clinical linear accelerator (photon average energy 1.6 MeV).

**Figure 5.**
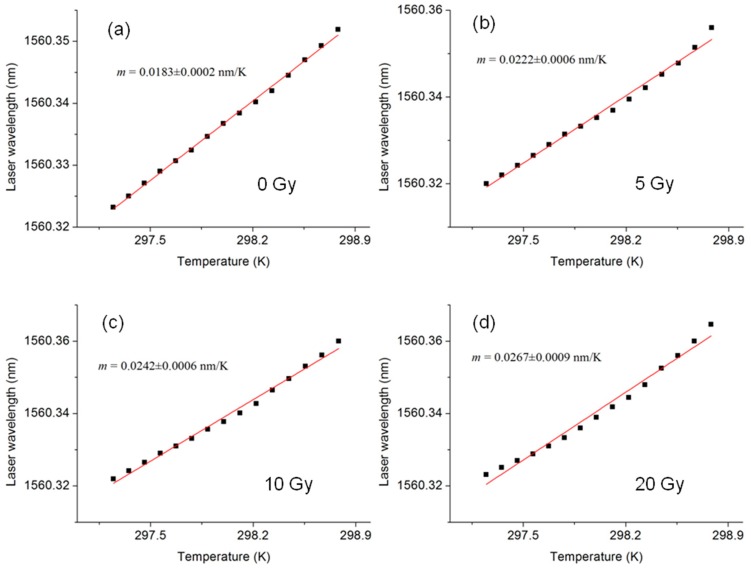
Wavelength of the laser locked to a cavity mode as function of the fiber temperature before irradiation (**a**) and after exposure to consecutive doses of 5 Gy, 10 Gy and 20 Gy (**b**–**d**). By a linear fit of the curves, we obtain the thermo-optic coefficient for each dose.

**Figure 6.**
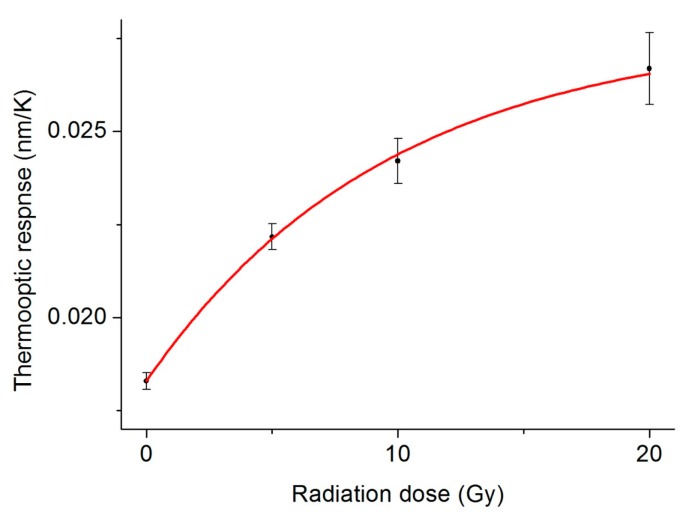
Thermo-optic response *versus* the dose delivered to the fiber. Saturation of the sensor response starts for doses higher than 10 Gy.

The fiber thermo-optic response was measured before irradiation and after exposure to gamma rays generated by a clinical linear accelerator (average energy 1.6 MeV) with consecutive doses of 5 Gy, 10 Gy and 20 Gy (Figure 4). The fiber was placed into a phantom made of Polymethylmethacrylate (PMMA), which is a good water-equivalent material. Figure 5a shows a graph of the cavity mode wavelength as function of the fiber temperature before irradiation. The data points are well approximated by a line whose angular coefficient *m* is the cavity thermo-optic coefficient. From a linear fit of the curve we obtain *m* = 0.0183 ± 2 × 10^−4^ nm/K. Figure 5b–d show the thermo-optic response measured after each dose, where a dependence of the slope of the curves on the dose is evident. In particular, the thermo-optic response for doses of 5 Gy, 10 Gy and 20 Gy were respectively 0.0222 ± 0.0006 nm/K, 0.0242 ± 0.0006 nm/K and 0.0267 ± 0.0009 nm/K. In Figure 6, the value of the thermo-optic response *versus* the dose delivered to the fiber is plotted. The curve exhibits an exponential behavior, showing the beginning of sensor saturation for doses above 10 Gy. From a single exponential fit, we obtain a saturation constant of about 9.6 Gy. Approximating the start of the exponential curve with a straight line, we obtain a linear trend with an angular coefficient of 0.0011 nm/K/Gy. Dividing this coefficient by the average value of the uncertainty on the single measurement of the thermo-optic response (0.0004 nm/K) we obtain a final detection limit of 360 mGy.

These results show that the sensor can be used in those contexts where high sensitivity in small volumes is required, but at the cost of a limited dose range. The most appropriate field of application would be medical dosimetry, where doses on the order of 1 Gy or less are typically involved in radiotherapy treatments.

## 4. Conclusions

In this work, we investigated the effects of IR on standard SMF28^TM^ Ge-doped fibers. A piece of fiber, enclosed between two FBGs, was irradiated with gamma rays from a clinical accelerator. We observed a change of the fiber thermo-optics response with the dose. We demonstrated that exploiting this effect the Ge-doped fiber can be used as IR sensor, obtaining a detection limit of 360 mGy. The experiment has been performed with a 2-cm long fiber, but, in principle, longer fibers can be used to increase the irradiated length and thus the sensitivity. For example, a 1-m long fiber could easily be arranged in a spiral over a surface of few cm^2^, leading to a detection limit of few mGy. As proof of principle we have chosen commercial Ge-doped fibers, but also other dopants could be considered. A systematic analysis of fiber materials and dopants, in order to further improve the system performance and sensitivity, will be the subject of a future work.

It is worth noting that the FBG optical cavity built around the fiber served only for measuring the thermo-optic response variations of the fiber, the FBGs being not irradiated at all. Thus, this configuration opens the way to a new class of very sensitive, cheap and disposable dosimeters based on doped fibers. The fiber could just be irradiated alone and then placed within a fiber cavity made up of two FBGs or a fiber-loop. In addition, dosimeters based on this method could be used both in small volumes or over large areas. The sensitive fiber could reach harsh and hardly accessible regions in a minimally invasive manner, such as in medical settings or industrial equipment. On the contrary, a grid of fibers could be placed over large areas, embedded in large structures or underground, e.g., in environmental monitoring and radio-protection applications.
